# Is the Current Systematic Subdivision of the Subfamily Neanurinae (Collembola, Neanuridae) Still Valid? Testing the Monophyly and Phylogenetic Relationships of Currently Established Tribes of the Neanurinae

**DOI:** 10.3390/insects15090672

**Published:** 2024-09-05

**Authors:** Grzegorz Paśnik, Adrian Smolis

**Affiliations:** 1 Institute of Systematics and Evolution of Animals, Polish Academy of Sciences, Sławkowska 17, 31-016 Kraków, Poland; pasnik@isez.pan.krakow.pl; 2 Institute of Environmental Biology, Department of Invertebrate Biology, Evolution and Conservation, University of Wrocław, Przybyszewskiego 65, 51-148 Wrocław, Poland

**Keywords:** taxonomy, classification, maximum parsimony, Bayesian Inference, characters evolution

## Abstract

**Simple Summary:**

The subfamily Neanurinae is the largest in the family, with almost 800 species. These springtails are different from all other Collembola in their appearance, behaviour and habitats. A division of Neanurinae into tribes was proposed by Cassagnau in 1989, but it has not yet been tested using cladistic methods. New studies suggest that the tribes currently recognised may not be monophyletic. A dataset of 101 discrete morphological characters was analysed to explore the phylogenetic relationships among major lineages of the Neanurinae. Bayesian and maximum parsimony analyses yielded similar tree topologies. The results indicate that the taxonomic characters used in the classification of Neanurinae are shared among members of the different tribes. The article discusses the phylogenetic significance of morphological characters, including those recognised as key to the evolution and history of Neanurinae.

**Abstract:**

The subfamily Neanurinae is the largest in the family, with almost 800 described species. These springtails differ significantly from all other Collembola in their morphology, behaviour, and natural habitats. A systematic division of the Neanurinae into tribes was proposed more than 30 years ago by Cassagnau (1989), but it has not yet been tested using cladistic methods. Recent studies, both phylogenetic analyses of individual tribes or genera and descriptions of new taxa, suggest that the currently recognised tribes may not be monophyletic. The phylogenetic relationships among major lineages of the Neanurinae were explored by analysing a dataset of 101 discrete morphological characters. Bayesian and maximum parsimony analyses yielded similar tree topologies. The relationships among the Neanurinae were not resolved in any of the analyses, except for the support for the monophyly of the tribe Lobellini. The results indicate that the taxonomic characters used in the classification of Neanurinae are shared among members of the different tribes, which may have resulted in a classification with little phylogenetic basis. The article discusses the phylogenetic significance of morphological characters, including those recognised as key to the evolution and history of Neanurinae.

## 1. Introduction

Springtails (Collembola) are one of the most primitive modern hexapods. Their well-preserved fossils date back to 400 million years ago. They are widespread, inhabiting regions from the equator to the poles, including the farthest reaches of the Arctic and Antarctic, and they are abundant, reaching densities of several thousand individuals per m^3^ of soil and leaf litter in temperate forests [1]. The Collembola class is typically divided into two subclasses: Arthropleona and Symphypleona *sensu lato* [1,2,3,4]. Arthropleona comprises two orders, Poduromorpha and Entomobryomorpha. The former includes up to one-third of all described species and genera of springtails [5]. Within Poduromorpha, the family Neanuridae is particularly noteworthy, as its members are known to occur on all continents, including Antarctica. This family is one of the largest and most species-rich, with over 1500 described species, which is one-sixth of all currently known Collembola [5].

Following the recent separation of the Odontellidae and the Brachystomellidae into separate families, the Neanuridae have traditionally been divided into six subfamilies: Frieseinae, Neanurinae, Pseudachorutinae, Morulininae, Caputanurininae, and Uchidanurinae. The latter two subfamilies have a limited number of species and are found only in eastern and southeastern Asia, Australia, New Zealand, and New Caledonia [1]. The subfamily Neanurinae is the largest in the family, with almost 800 described species. These springtails differ significantly from all other Collembola in their morphology, behaviour, and natural habitats. Above all, they have completely lost the furcula, typical of most Collembola, and their movements can be described as exceptionally slow in comparison with the vast majority of this class. One of the differences between the Neanurinae and most of the other Collembola is the presence of spherical tubercles on the dorsal surface of the body, which gives them a certain resemblance to mulberries. Additionally, the chaetae covering the body of Neanurinae are typically well-developed, elongated, broad, and equipped with numerous teeth. Despite their slow movements and lack of the furcula structure for evading predators, Neanurinae are considered an evolutionary success. This is evidenced by the fact that they make up nearly one-tenth of all known Collembola taxa [5]. 

Several factors have contributed to this success, with three appearing to be the most critical. Firstly, the tubercles and stiff chaetae covering the body create a crucial mechanical barrier against predators. Secondly, this distinctive mode of defence is reinforced by the production of volatile poisonous chemical substances, such as phenols [6,7]. The third characteristic is their narrow trophic specialisation, with slime moulds being their preferred source of food. This has only recently been observed and confirmed experimentally [8,9,10,11]. Slime moulds are single- or multi-celled, depending on the stage of development, and are found primarily in very moist terrestrial habitats. The same type of habitat is also preferred by the Neanurinae, which predominantly occur in forest ecosystems, with tropical and temperate woodlands being particularly rich in Neanurinae species. 

Despite the significant scientific interest in the Neanurinae subfamily, its classification and understanding have undergone numerous modifications since its establishment in 1901 by Börner [12]. In 1981, Deharveng [13] analysed the dorsal side of the fourth antennal segment in various Neanuridae members and identified a consistent and distinct arrangement of certain setae in their chaetotaxy. Since that time, this character has become the most important and least controversial criterion for determining the membership of the subfamily. In the late 1980s, Cassagnau [4] proposed dividing Neanurinae into six tribes: Morulodini, Neanurini, Lobellini, Paranurini, Paleonurini, and Sensillanurini. This classification is based on a combination of the following characters: the number of eyes and their colour, the colour of the cuticle, the degree of reduction of the mouthparts, the degree of development of the tubercles and the size of the antennal sensilla. Cassagnau proposed a new division of Neanurinae and described the biogeography of this subfamily, including the centres of differentiation and the directions of expansion of the individual tribes. The author identified trends in the development of specific characters and found that the most significant evolutionary changes in Neanurinae were the gradual reduction and simplification of some components of the mouthparts, such as the mandibles and maxillae, and an increasing degree of tuberculisation, which refers to the transition from forms completely devoid of these structures, through forms with only a few tubercles on certain segments of the body, to advanced forms characterised not only by tubercles covering all segments but also by their combination and fusion within these segments. For the past 30 years, Cassagnau’s proposed classification system and scheme of evolution for this family has been widely accepted and applied.

It is noteworthy that, to date, this system has not undergone a critical analysis using cladistic tools. Previous phylogenetic studies of Neanurinae have typically concentrated on individual genera or a small number of species from a maximum of two of the recognised tribes. For example, molecular studies based on nuclear rRNA 28S and the mitochondrial gene COII have highlighted the monophyly of two tribes, Paleonurini and Neanurini [14]. However, it is important to note that this study used only one genus from the former tribe, *Bilobella* Caroli, 1912 [15], and that this genus included three closely related species (*B. aurantiaca* Caroli, 1912, *B. braunerae* Deharveng, 1981 and *B. massoudi* Cassagnau, 1968) [15,16,17]. In contrast, a phylogenetic study based on 380 different cuticular lipids placed *Bilobella aurantiaca* among the members of Neanurini, which contradicts the results of molecular analyses [18]. Interestingly, cladistic analysis of the genus *Palmanura* Cassagnau, 1983 (Sensillanurini) [19], in which outgroup taxa were represented by two species from the tribes Paranurini and Neanurini, did not fully support the monophyly of this tribe [20]. A recent study by Smolis and Paśnik [21] analysed the phylogeny of Neanurini, the second-largest tribe within Neanurinae. This study used species belonging to 25 of the tribe’s 29 genera. Representatives from each of the other tribes were included in the study to validate the classification of genera within the tribe. This analysis questions the monophyly of up to four of the six tribes proposed by Cassagnau. Therefore, Cassagnau’s system requires critical and rigorous analysis using cladistic tools. 

In view of the above aspects, which call into question the validity of the current system of classification of this subfamily, which includes up to 10% of all described Collembola, a cladistic analysis was carried out using a suitable sample of species and genera assigned within the currently accepted classification system. The main objectives of the analysis were to (1) verify and validate the monophyly of the tribes established in Cassagnau’s system, (2) analyse the phylogenetic relationships between the different tribes, and (3) discuss the phylogenetic significance of morphological characters, including those recognised as key to the evolution and history of Neanurinae.

## 2. Material and Methods

### 2.1. Taxon Sampling

To evaluate the monophyly and relationships among the currently recognised tribes of Neanurinae, we analysed 38 terminal taxa (Table 1). Genera were selected based on species availability, with a preference for species type. We included seven genera from the tribe Lobellini, one genus from the tribe Morulodini, ten genera from the tribe Neanurini, twelve genera from the tribe Paleonurini, three genera from the tribe Paranurini, and five genera from the tribe Sensillanurini. The analysis includes only one taxon representing Morulodini. Although several species of the genus *Morulodes* Cassagnau, 1955 [22] have been described, their original descriptions lack most of the characters used in this paper and were therefore excluded from the analyses. The trees were rooted using *Friesea mirabilis* (Tullberg, 1871) (subfamily Frieseinae) and *Pseudachorutes palmiensis* Börner, 1903 (subfamily Pseudachorutinae) [23,24] as outgroup taxa.

### 2.2. Morphological Data

This study aimed to consider morphological variation within each genus, especially for tribes from different geographical regions. The characters used were based on personal observations of specimens, supplemented by previous taxonomic and phylogenetic studies [8,25,26,27,28,29,30,31,32,33,34].

A total of 101 characters were scored for the study taxa, including 65 binary characters and 36 multistate characters. The list of characters is available in Supplementary Table S1. Missing data were coded as ‘?’ in the matrix (Supplementary Table S2). All characters were treated as unordered [35] and equally weighted [36], thus making no assumptions regarding character evolution. Autapomorphies were retained in the data matrix [37], as they might become synapomorphies when new taxa are described, and taxon sampling improves, but were deactivated for the calculation of the ensemble value of the consistency index (CI) as proposed by Bryant [38].

A character matrix was constructed, and characters were mapped using WinClada ver. 1.00.08 [39] to observe the character state transformation on a tree.

The specimens were examined using a Zeiss Axio Imager (Carl Zeiss Microscopy GmbH, Oberkochen, Germany), an A2 compound microscope (Carl Zeiss Microscopy GmbH, Oberkochen, Germany), and a Nikon Eclipse E600 phase contrast microscope (Nikon Europe B.V., Amstelveen, The Netherlands). The morphological terminology used is largely based on Deharveng (1983) [40], Deharveng and Weiner (1984) [41], Greenslade and Deharveng (1989) [42], Lawrence (1977) [43] and Smolis (2008) [44].

**Table 1:** Species examined together with their geographical distribution. Geographical distribution of species is indicated by region: 1, Nearctic; 2, Palaearctic; 3, Afrotropical; 4, Oriental; 5, Australasian (including western Pacific Islands); 6, Neotropical; 7, Cosmopolitan.

Tribe	Genus	Species	Distribution
Lobellini	*Coecoloba*	*plumleyi Deharveng*, 1983 [40]	5
	*Hemilobella*	*rounsevelli* Deharveng & Greenslade, 1992 [28]	5
	*Lobellina*	*weinerae* Smolis, 2017 [45]	4
	*Paralobella*	*breviseta* Luo & Palacios-Vargas, 2016 [33]	4
	*Sulobella*	*yoshii* Deharveng & Suhardjono, 2000 [31]	4
	*Telobella*	*kemiri* Suhardjono & Deharveng, 2001 [46]	4
	*Yuukianura*	*judithae* Deharveng, Palacios-Vargas & Bedos, 2017 [8]	5
Morulodini	*Morulodes*	*serratus* (Folsom, 1916) [47]	1
Neanurini	*Deutonura*	*phlegrea* (Caroli, 1912) [15]	2
	*Edoughnura*	*rara* Deharveng, Hamra-Kroua & Bedos, 2007 [30]	2
	*Ghirkanura*	*chernovae* Kuznetzova & Potapov, 1988 [32]	2
	*Intricatonura*	*fjellbergi* Smolis & Bernard, 2017 [48]	1
	*Monobella*	*grassei grassei* (Denis, 1923) [49]	2
	*Neanura*	*muscorum* (Templeton, 1835) [50]	7
	*Paravietnura*	*notabilis* Smolis & Kuznetzova, 2018 [51]	2
	*Thaumanura*	*carolii* (Stach, 1920) [52]	2
	*Vietnura*	*caerulea* Deharveng & Bedos, 2000 [53]	4
	*Xylanura*	*oregonensis* Smolis, 2011 [54]	1
Paleonurini	*Australonura*	*grossi* (Yosii, 1966) [55]	5
	*Bilobella*	*carpatica* Smolis & Kaprus’, 2008 [56]	2
	*Caledonura*	*tillierae* Deharveng, 1988 [57]	5
	*Cameronura*	*delamarei* Cassagnau, 1991 [27]	3
	*Ectonura*	*lata* Deharveng, Weiner & Najt, 1997 [29]	5
	*Galanura*	*agnieskae* Smolis, 2000 [58]	2
	*Himalmeria*	*gurung* Cassagnau, 1984 [26]	4
	*Itanura*	*brasiliensis* Queiroz & Deharveng, 2015 [59]	6
	*Paleonura*	*epiphytica* Smolis & Deharveng, 2003 [60]	4
	*Pronura*	*pomorskii* Smolis & Deharveng, 2006 [61]	4
	*Vitronura*	*mascula* Smolis & Deharveng, 2006 [62]	4
	*Zelandanura*	*bituberculata* Deharveng &Wise, 1987 [63]	5
Paranurini	*Nahuanura*	*ce* Palacios-Vargas & Najt, 1986 [34]	1
	*Oregonanura*	*cascadensis* Smolis, 2008 [44]	1
	*Paranura*	*sexpunctata* Axelson, 1902 [64]	1, 2
Sensillanurini	*Americanura*	*mexicana* Cassagnau, 1983 [19]	1
	*Honduranura*	*centraliamericana* Palacios-Vargas, 2017 [65]	1
	*Palmanura*	*mirabilis* Cassagnau & Palacios-Vargas, 1983 [25]	1
	*Sensillanura*	*austriaca* (Gama, 1963) [66]	2
	*Tabasconura*	*tapijulapana* Palacios-Vargas & Catalán, 2015 [67]	6

### 2.3. Phylogenetic Analysis

Two methods were chosen to examine different approaches to the reconstruction of evolutionary relationships: Maximum Parsimony (MP) and Bayesian Inference (BI).

Parsimony analyses using both equal and implied weights were performed using TNT version 1.6 [68]. To find the most parsimonious trees, the analyses were run with the ‘New Technology Search’ option [69] with the following parameters: general RAM of 3000 Mbytes, memory set to hold 500,000 trees, and zero-length branches collapsed. The searches consisted of Tree Fusion, Ratchet, Tree Drifting, and Sectorial searches performed, with default parameters applied, until the most parsimonious tree was found 100 times. All characters were treated as unordered and equally weighted. 

The search for the most parsimonious trees was performed by first applying equal weights to all characters and subsequently applying implied weights. It has been argued e.g., [70,71] that it is preferable to obtain results using the correct weighting of characters rather than using the same weighting for all characters. Implied weighting is a commonly used method for assigning different weights during a tree search. It is a good choice because it is independent of any previous analyses and of any previous weights. The strength against homoplasy under implied weighting is related to a constant, k. A lower value of k indicates a higher strength against homoplasy. This value represents the ratio of a single additional step to the cost of the most homoplasious character. The value of k was calculated using the TNT script setk.run written by Salvador Arias (Instituto Miguel Lillo in San Miguel de Tucuman, Argentina), which returned a value of 7.187500 for our dataset. The implied weight searches used the same parsimony options.

Clade support was evaluated using symmetric resampling [72]. Symmetric Resampling (SR) support measures the difference in frequencies between a given group and its most frequent contradictory group (GC). The analyses were performed in TNT using the traditional search method with 10,000 replications, a change probability of 0.33, two initial Wagner trees, and three trees held per replicate.

Bayesian inferences were performed in MrBayes v3.2.5 [73] using two simultaneous Markov Chain Monte Carlo runs, with 4 chains of 10 million generations each, sampling trees every 1000th generation. For this analysis, the dataset was treated as a single partition and analysed using gamma distribution variation. All state frequencies (change rates) were set equal, all topologies had equal probabilities, and the branch length was unconstrained. Posterior probabilities (PP) were interpreted as statistical support values for the tree resulting from Bayesian inference.

The following values were applied to support the clades: weak, SR < 50%; moderate, SR 51–75%; good, SR 76–90%; and strong, SR > 90%.

## 3. Results

**Figure 1:**
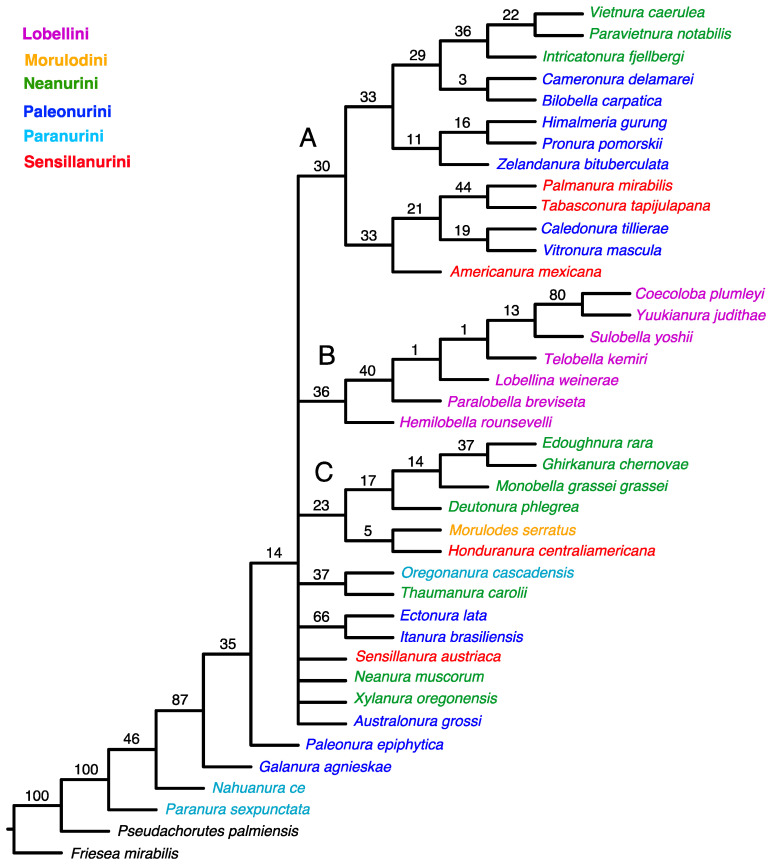
Strict consensus from MP analysis using unweighted characters. Values for GC frequencies (symmetric resampling) are shown above branches. The main clades are indicated by letters (A–C).

An equal-weight analysis yielded the 51 most parsimonious trees with 510 steps, consistency index (CI) = 0.278, and retention index (RI) = 0.502. This strict consensus indicates a lack of resolution among the taxa studied (Figure 1).

**Figure 2:**
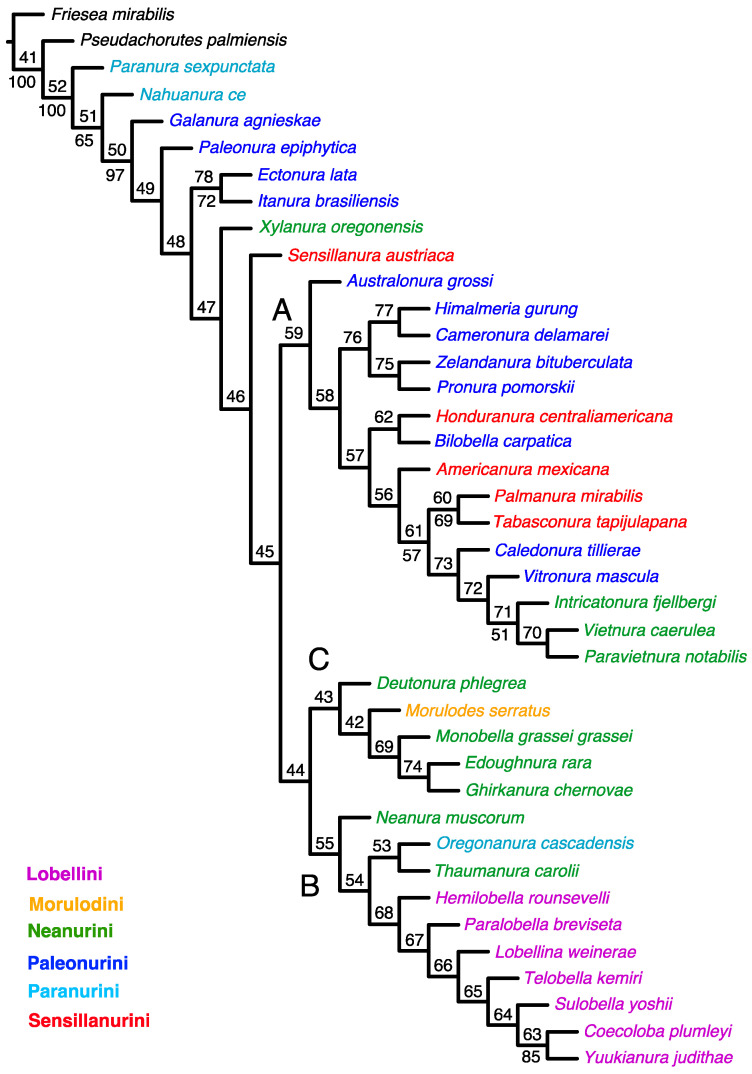
Single cladogram obtained in the analysis of morphology under implied weights k = 7.187500 (length = 514; fit = 27.02090). Node numbers are shown above the branches, and GC frequencies (symmetric resampling) are shown below the branches. Only values above 50 are indicated to facilitate the visualisation of the most internal branches. The main clades are indicated with letters (A–C).

The implied weighting analysis retrieved the most parsimonious cladogram with *k* = 7.187500, best score = 27.02090, steps = 514, consistency index (CI) = 0.288, and retention index (RI) = 0.525 (Figure 2). 

**Figure 3:**
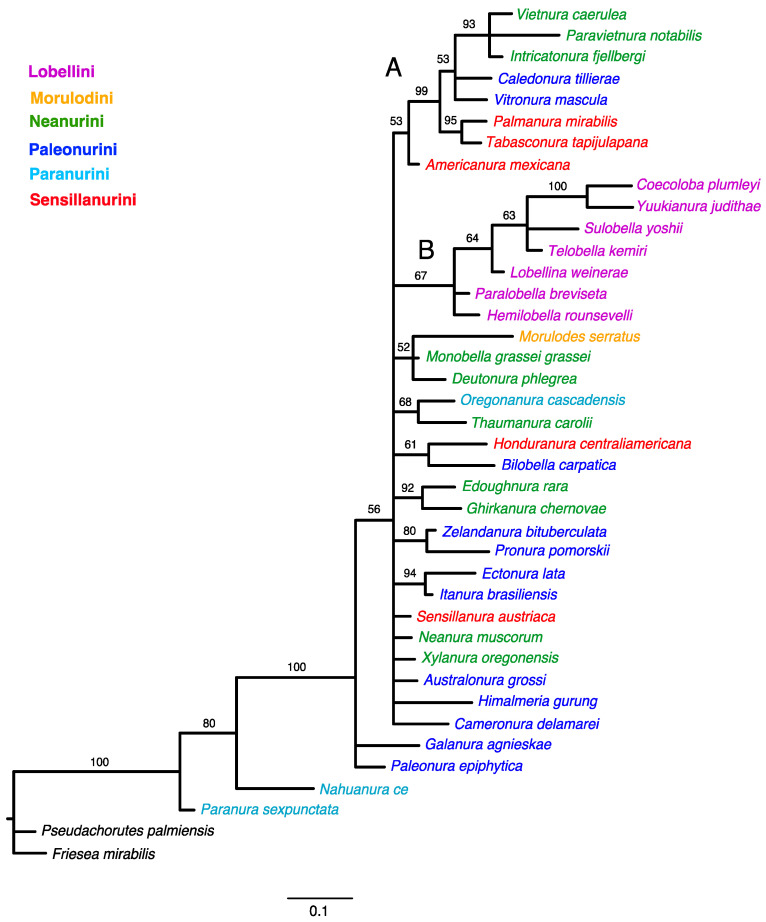
Phylogenetic trees inferred from Bayesian Inference (BI). Numbers above branches are Bayesian posterior probability (PP) values (>50). The main clades are indicated with capital letters on the branches.

Bayesian analysis of the morphology dataset achieved stationarity after ten million generations when an average standard deviation of split frequencies has fallen below 0.01 (0.008150). The 50% majority-rule consensus tree of the post-burn-in posterior distribution is shown in Figure 3. 

A list of the morphological apomorphies for each resolved node on the tree shown in Figure 2 is provided in Supplementary Table S3. 

The implied weighting tree provided better resolution than the Bayesian and equal weighting topologies. The relationships of Neanurinae were not resolved in any of the analyses, with the exception of support for the monophyly of Lobellini. While several groups were identified, the taxa within these groups did not correspond to the current systematic classification and did not have high support (both PP for Bayesian and SR for maximum parsimony analyses).

The tree topology from both the equal-weighted and Bayesian analyses was mostly unresolved. The equal-weighted analysis grouped most taxa into three clades (Figure 1), of which two corresponded to similar groupings in the Bayesian tree (Figure 3). Clade ‘A’ (Figure 1) comprises 13 genera representing the tribes Neanurini, Paleonurini and Sensillanurini, but this relationship lacks support. The Bayesian tree’s corresponding clade (clade ‘A’ in Figure 3) comprises only eight of these genera. With the exception of the basal taxon (*Americanura mexicana* Cassagnau, 1983), this clade, which includes representatives of three different tribes, has strong support (PP–99). 

In both analyses, clade ‘B’ (Figure 1 and Figure 3) has the same topology and includes all studied genera of the tribe Lobellini, but the monophyly of the tribe has no support in the parsimony analysis (SR–36) and only moderate support in the Bayesian analysis (PP–67). 

Four representatives of Neanurini, one genus of Sensillanurini (*Honduranura centraliamericana* Palacios-Vargas, 2017), and a single member of Morulodini are grouped together in clade ‘C’ (Figure 1). However, this clustering method lacks support and does not appear in the Bayesian tree. 

The tree topology resulting from the implied weighting analysis (Figure 2) is comparable to that of the tree from the equal weighting analysis. Similarly, there are three clades, but their composition and arrangement of taxa differ. Clade ‘A’ comprises all the species present in the tree from the equal weighting analysis, as well as *Australonura grossi* (Yosii, 1966) (Paleonurini) and *Honduranura centraliamericana* Palacios-Vargas, 2017 (Sensillanurini). The latter genus is included in clade ‘C’ of the parsimonious tree. Clade ‘B’ comprises all studied representatives of the tribe Lobellini and the two Neanurini genera (*Neanura muscorum* (Templeton, 1835) and *Thaumanura carolli* (Stach, 1920)) and one representative of the tribe Paranurini (*Oregonanura cascadensis* Smolis, 2008). Clade ‘C’ includes four genera of the tribe Neanurini and a single genus of the tribe Morulodini. All clades and groupings within clades have very low or no support at all.

## 4. Discussion

### 4.1. Taxonomy

The classification system for Neanurinae proposed by Cassagnau [4], dividing them into six tribes: Morulodini, Neanurini, Lobellini, Paranurini, Paleonurini, and Sensillanurini, has been widely accepted and used for more than three decades, but our cladistic analyses (Figure 1, Figure 2 and Figure 3) have significantly questioned this system. 

The doubts and reservations about this classification have been highlighted by several studies: the cladistic study of Neanurini [21] and the genus *Palmanura* [20], the phylogenetic study based on cuticular chemistry [18], and several taxonomic papers describing new genera (e.g., [32,44,54,56]). 

The cladistic analysis supports previously published reservations and indicates that the current system of classifying Neanurinae into tribes should be revised. Out of the six tribes proposed by Cassagnau [4], only Lobellini has been confirmed to be monophyletic (Figure 1). It is important to note that Cassagnau emphasised the importance of biogeographic data in phylogeny reconstruction when proposing the classification tested in this paper. Although Lobellini is widely distributed, most of its constituent species and genera are restricted to the western Pacific region, including eastern and southeastern Asia, Australia and New Guinea, the Solomon Islands, New Caledonia and the Hawaiian Islands. The distribution of this tribe is disrupted only by species that have been introduced to other parts of the world by humans. For example, members of the genus *Yuukianura* Yosii, 1955 [74] were discovered at an earthworm farm in Great Britain [75], and two species have been described from eastern North America [76] and Cuba [77]. Analysis of contemporary materials has shown that *Lobella palmeri* (Wray, 1967) [76] possesses unique characters not found in other Lobellini species, casting doubt on its membership in this tribe (Smolis, A., and Bernard, E.C. (*manuscript in preparation*). Therefore, the distinctiveness of Lobellini is supported by both cladistic and biogeographical data. 

The single genus and species analysed from the tribe Morulodini, *Morulodes serratus* Folsom, 1916, was grouped with representatives of the Neanurini. Its monophyly and systematic position within Neanurinae remains uncertain due to the limited representation caused by the lack of study material.

The majority of the genera belonging to the tribes Paleonurini and Sensillanurini were grouped together within the clade designated as ‘A’ (Figure 2). The former are present on all continents (with the exception of Antarctica), while the Sensillanurini are mainly found in the New World region. An exception among the latter is *Sensillanura austriaca* (Gama, 1963), which has been documented only in a limited area of Europe and North Africa [13]. Notably, in our analysis, this species did not occur alongside the other representatives of the aforementioned tribes; rather, it formed a sister group to clades ‘A’ and ‘B’ (Figure 2).

As previously stated, representatives of the tribe Paleonurini do not form a distinct monophyletic group. This most widely distributed tribe in the subfamily Neanurinae is also among the most morphologically diverse. For this reason, among others, Cassagnau [40,78] proposed the existence of three distinct lineages within the Palaeonurini, namely the “lignée blasconurienne, bilobellienne et phyliomerienne”. It is noteworthy that taxa from the aforementioned lineages are sometimes grouped together and that they are often taxa from other zoogeographical realms. For example, *Ectonura lata* Deharveng, Weiner & Najt, 1997 and *Itanura brasiliensis* Queiroz & Deharveng, 2015 (both taxa from the “blasconurienne” lineage, Figure 1, Figure 2 and Figure 3), or *Himalmieria gurung* Cassagnau, 1984 and *Cameronura delamarei* Cassagnau, 1991 (from the “phyliomerienne” lineage, Figure 2). The aforementioned clustering of taxa within the obtained clades may be indicative of closer relationships or of an ancient origin of these lineages, potentially extending back to the time of Gondwana, as previously proposed by Cassagnau [19]. The remaining Palaeonurini, *Galanura agnieskae* Smolis, 2000, and *Palaeonura epiphytica* Smolis & Deharveng, 2003 consistently occupy positions at the base of the Neanurinae tree (Figure 1, Figure 2 and Figure 3).

Representatives of the tribe Neanurini were scattered throughout all three clades. The genera *Deutonura*, *Monobella*, *Edoughnura* and *Ghirkanura*, in conjunction with *Morulodes*, constitute a distinct group that is evident in the parsimony analysis (Figure 1 and Figure 2, clade C). However, this group is not present in the Bayesian tree. This may be attributed to the fact that these taxa exhibit a number of shared features, such as chaetotaxy and arrangement of the posterior tubercles, which are not present in the majority of the other taxa included in the analysis.

Three additional genera (*Vietnura* Deharveng & Bedos, 2000; *Intricatonura* Smolis & Bernard, 2017; and *Paravietnura* Smolis & Kuznetsova, 2018) clustered together within clade A in all analyses. It is noteworthy that these three taxa are grouped together with two representatives of the Palaeonurini (genera *Caledonura* and *Vitronura*) and two Sensillanurini (*Palmanura* and *Tabasconura*) to form a highly supported group on the Bayesian tree (PP–99) (Figure 3) and a moderately supported group on the implied weighted analysis tree (SR–57). The close phylogenetic relationships between *Vietnura* Deharveng & Bedos, 2000, *Intricatonura* Smolis & Bernard, 2017, and *Paravietnura* Smolis & Kuznetsova, 2018, were also obtained in the phylogenetic analysis of the tribe Neanurini [21]. It is particularly noteworthy that these taxa have been described from very distant locations. These include Southeast Asia (Vietnam and southern China) [53,79], eastern North America [48], and the Caucasus [51]. Consequently, their morphological similarities are regarded as the result of convergence rather than true relationships [48,51]. The results obtained may indicate a close relationship between these taxa, which is further supported by their occurrence in known tertiary refugia, as illustrated in Figure 1 in Milne and Abbott [80].

Two other genera from the tribe Neanurini, *Neanura* and *Xylanura*, consistently fell outside the groupings observed in the analyses. They occupy separate positions. This may be particularly surprising with regard to *Neanura muscorum* (Templeton, 1835), which is the type species for the tribe and the subfamily as a whole. However, this situation can be relatively easily explained by the evident morphological distinctiveness of this species, manifested by a number of features. These include a higher number of eyes, the arrangement of Di and De chaetae in the posterior part of the head, which is rare within the tribe, or the unique arrangement of tubercles in its lateral parts. *Xylanura oregonensis* Smolis, 2011 exhibits the first two of the aforementioned features, in addition to a reduction in tubercles, which brings it into close proximity with taxa from the tribes Paleonurini and Paranurini, which manifest a similar tendency to completely or partially lack these structures. This taxon has features that are relatively uncommon within the Neanurini (for example, as seen in *N. muscorum*) or that are exclusive to other tribes. The last member of Neanurini (*Thaumanura*) is combined with *Oreganura* on all trees. The reason for this may be sought in the presence of a unique feature, namely the elongation of the fifth segment of the abdomen. 

The two other members of the Paranurini, namely *Nahuanura ce* Palacios-Vargas & Najt, 1986 and *Paranura sexpunctata* Axelson, 1902, are consistently positioned at the base of trees. Both are characterised by the complete absence or highly incomplete tuberculation of the body. It is noteworthy that three additional taxa exhibiting incomplete tuberculation, *Paleonura epiphytica* and *Galanura agnieskae* (both from the tribe Paleonurini) and *Xylanura* (from the tribe Neanurini), are also located outside the clades (Figure 1, Figure 2 and Figure 3). This positioning of the above taxa is of interest in the context of Cassangnau’s [4,78] consideration of the evolution of the subfamily Neanurinae. Furthermore, in addition to the classification of Neanurinae, this researcher presented a probable scenario for the origin and subsequent expansion routes of the individual tribes. In essence, he postulated that the ancestors of the entire subfamily were representatives of the subfamily Pseudachorutinae, classified in the Holarctic genus *Anurida*, specifically in the group of species termed *Anurida gr. hammerae*. The species in question are found in a limited geographical region, namely north-eastern Russia and north-western North America (including north Canada, Alaska, and Washington in the USA). These species display a number of characteristics typically seen in members of the subfamily Neanurinae, namely the displacement of p2 and p3 chaetae on the thorax anteriorly, the reduction of axial chaetotaxy, or the presence of reticulation but absence of tubercles. 

According to Cassagnau’s hypothesis, representatives of the various tribes of Neanurinae, which are characterised by the complete or partial absence of tubercles, the so-called “Neanurinae Paucitubercles”, are therefore considered to be among the most primitive within the various tribes and evolutionary lineages. This hypothesis is supported to some extent by the results obtained.

### 4.2. Morphological Characters

Recent work has shown that certain characters previously used by Cassagnau for the differentiation of the six tribes are not entirely diagnostic and should, therefore, no longer be used for their differentiation or definition. For instance, *Ghirkanura chernovae* Kuznetsova & Potapov, 1988, a member of the tribe Neanurini, was found to have a single-lobed end of the abdomen, typical and characteristic of the tribe Paranurini (in the other Neanurinae, the end of the abdomen is double-lobed). The tribe Sensillanurini is characterised by hypertrophy of the seventh sensillum on the fourth antennal segment, a character described in *Galanura agnieskae* Smolis, 2000, a member of Paleonurini. However, the recent discovery of *Oregonanura cascadensis* Smolis, 2008, a member of Paranurini, with fully developed tubercles on the dorsal body surface, challenges the previous assumption regarding the primitiveness of this tribe in Cassagnau’s phylogenetic scenario of Neanurinae. According to this scenario, members of Paranurini, such as those of the genus *Paranura* Axelson, 1902 without tubercles, should be considered the most primitive. In addition, the recently discovered neanurine springtail, *Xylanura oregonensis* Smolis, 2011, lacks tubercles on the first thoracic segment. Therefore, incomplete tuberculisation may not only occur in Paranurini and Paleonurini, the lineages that Cassagnau considered to be the closest ancestors of Neanurinae. These recent discoveries have not only broadened our understanding of the diversity of this subfamily worldwide but have also removed certain diagnostic characters from certain lineages, providing compelling evidence and highlighting the need for new analyses and revisions of the phylogenetic relationships within this large subfamily of Collembola. 

Furthermore, the Cassagnau classification system, as well as other classification proposals for Neanurinae, are typically based on a severely limited number of subjectively selected features. The preceding analysis has demonstrated that this approach may result in the exclusion of a number of phylogenetically relevant and useful characters from the analysis. For instance, clade A was distinguished based on the status of certain characters, including the number of labial and prelabral chaetae, number of tubercles on the last segment of the abdomen, and presence of the tibiotarsal bristle M. In contrast, clade C, which exhibited the highest rate of support, was distinguished based on the fusion and chaetotaxy of the cephalic tubercles Di and De, as well as the shape and length of the abdomen macrochaetae. The aforementioned characters have never been considered for classification at a higher level than the generic. This evidence demonstrates that subfamily divisions into tribes based on a limited set of features are highly incomplete and biased by design.

## 5. Conclusions

The results of the analysis appear to challenge, if not overturn, the systematic division that has been widely accepted and applied by many researchers in this highly diverse and species-rich subfamily of Collembola. However, the history of the classification of Neanurinae is replete with classification proposals that are controversial from today’s point of view. For example, the classification of the genus *Paranura* within another subfamily, Pseudachorutinae, or the inclusion of the genus *Morulina* almost from the very beginning of Neanurinae. Today, the latter constitutes, together with the monotypic genus *Promorulina*, a separate subfamily, Morulininae. It is noteworthy that a classification analogous to the outcomes of the present analyses was proposed in 1961 by Yosii [81], who distinguished three tribes within Neanurinae: Morulini, Neanurini, and Lobellini. Consequently, after excluding the aforementioned Morulini, only two tribes remain within the system proposed by Yosii: Neanurini and Lobellini. The latter, despite their relatively weak support, emerge as a monophyletic group in the analyses carried out in this paper (Figure 1 and Figure 3). The elevation of the remaining clades, designated A and C (Figure 1, Figure 2 and Figure 3), to the status of tribes at this stage of research is not adequately justified. In order to resolve these questions, further studies, including molecular methods, are required in our opinion. The combination of the latter with morphological data would provide the most comprehensive understanding of the relationships within Neanurinae.

## Data Availability

All data generated or analysed during this study are included in this published article and its supplementary information files. The datasets generated and/or analysed during the current study are also available from the corresponding author upon reasonable request.

## Supplementary Materials

The following supporting information can be downloaded at https://www.mdpi.com/article/10.3390/insects15090672/s1, Table S1: List of 101 characters for 38 taxa in the subfamily Neanurinae Börner, 1901 sensu Deharveng, 1981 and two outgroup taxa. Table S2: Morphological data matrix used in the analyses. Table S3: Morphological apomorphies. Character number from “Character list” followed by state in parentheses; non-homoplasious changes indicated in bold.
