# Supplementary material for: Is the Current Systematic Subdivision of the Subfamily Neanurinae (Collembola, Neanuridae) Still Valid? Testing the Monophyly and Phylogenetic Relationships of Currently Established Tribes of the Neanurinae
Source: Insects. 2024 Sep 5;15(9):672. doi: 10.3390/insects15090672 (PMC11432141; doi:10.3390/insects15090672)
Supplement: Supplementary file 1 [file insects-15-00672-s001.zip › Table S1. List of characters.pdf]

Table S1. List of 101 characters for 38 taxa in the subfamily Neanurinae Börner, 1901 *sensu* Deharveng, 1981 and two outgroup taxa.

No.	Morphological Characters and States
0.	Head – number of eyes on half of head: (0) 8–6 eyes present; (1) 5–4 eyes present; (2) 3 eyes present; (3) 2–0 eyes present.
1.	Head – pigmentation of eyes: (0) present; (1) absent.
2.	Head – number of lateral tubercles (Dl, L and So): (0) tubercles absent; (1) 1 tubercle present; (2) 2 tubercles present; (3) 3 tubercles present.
3.	Head – tubercles Dl and L: (0) absent; (1) present and separated; (2) present and fused.
4.	Head – tubercle L and So: (0) absent; (1) present and separated; (2) present and fused.
5.	Head – tubercles An and Fr: (0) absent; (1) present.
6.	Head – tubercle Af: (0) absent; (1) present; (2) present and divided along midline.
7.	Head – tubercle Cl: (0) absent; (1) present; (2) present and fused with tubercle Af.
8.	Head – tubercles Oc: (0) absent; (1) present; (2) present and fused with tubercle Af.
9.	Head – tubercles Af, Cl and Oc: (0) absent; (1) present and separated; (2) present and fused together.
10.	Head – tubercles De: (0) absent; (1) present.
11.	Head – tubercles Di and De: (0) absent; (1) present and separate; (2) present and fused.
12.	Head – tubercles Di: (0) absent; (1) present; (2) present and fused along midline.
13.	Head – chaetae A: (0) absent; (1) present and shorter than chaetae B; (2) present and equal chaetae B.
14.	Head – chaeta O: (0) present and not shifted; (1) present and shifted shifted towards tubercle Cl; (2) absent.
15.	Head – chaetae C: (0) present; (1) absent.
16.	Head – chaetae D: (0) absent; (1) present and free (not included in tubercle Af or Cl); (2) present and included in tubercle Af or An; (3) present and included in tubercle Cl; (4) present and included in tubercle (Af+Cl).
17.	Head – chaetae E: (0) present; (1) absent.
18.	Head – chaetae Oca: (0) present; (1) absent.
19.	Head – chaetae Ocp: (0) present; (1) absent.
20.	Head – chaetae sd2: (0) present; (1) absent.
21.	Head – chaeta a0: (0) present; (1) absent.
22.	Head – number of chaetae Di and De (on half of the head): (0) more than 4 chaetae present; (1) 4 chaetae present; (2) 3 chaetae present; (3) 2 chaetae present.
23.	Head – chaetae Di2: (0) present; (1) absent.
24.	Head – chaetae De2: (0) absent; (1) present and situated above chaetae De1; (2) present and situated below chaetae De1.
25.	Head – PAO: (0) present; (1) absent.
26.	Head – microsensillum on antenna IV: (0) present; (1) absent.
27.	Head – sensillum S5 on antenna IV: (0) absent; (1) present.

28. Head – sensillum S6 on antenna IV: (0) absent; (1) present.
29. Head – hypertrophy of sensillum S2 on antenna IV: (0) absent; (1) present.
30. Head – hypertrophy of sensillum S7 on antenna IV: (0) absent; (1) present.
31. Head – hypertrophy of sensillum S8 on antenna IV: (0) absent; (1) present.
32. Head – sensillum sgd on antenna III: (0) not migrated distally; (1) migrated distally, at level of sensilla S1 and S2 on antenna IV.
33. Head – ventral field on antenna IV: (0) present; (1) absent.
34. Head – buccal cone: (0) short and truncated; (1) elongated and beak-shaped.
35. Head – labial papillae x: (0) present; (1) absent.
36. Head – labial chaeta B: (0) present; (1) absent.
37. Head – labial chaeta D: (0) present; (1) absent.
38. Head – labial chaeta E: (0) present; (1) absent.
39. Head – labial chaeta f: (0) present; (1) absent.
40. Head – number of labial lateral chaetae: (0) 5 chaetae present; (1) 4 chaetae present; (2) 3 or less chaetae present.
41. Head – shape of labral apex: (0) truncate; (1) rounded; (2) ogival.
42. Head – number of prelabral chaetae: (0) 4 chaetae present; (1) 2 chaetae present; (2) chaetae absent.
43. Head – number of labral chaetal rows: (0) 3 rows; (1) 2 rows.
44. Head – number of chaetae on the first labral row: (0) 3 chaetae; (1) 2 chaetae; (2) chaetae absent.
45. Head – number of chaetae on the second labral row: (0) 5 chaetae; (1) 4 chaetae; (2) 3 chaetae; (3) 2 chaetae.
46. Head – number of chaetae on the apical labral row: (0) 4 chaetae; (1) 2 chaetae.
47. Head – shape of maxilla: (0) sickle-shaped; (1) styliform.
48. Head – maxilla with dentate lamella: (0) present; (1) absent.
49. Head – number of mandibular teeth: (0) more than 4 teeth; (1) no more than 4 teeth.
50. Head – mandibular lamellae: (0) present; (1) absent.
51. Body – color in alive: (0) blue or bluish grey; (1) orange or yellow; (2) white.
52. Body – shape of dorsal macrochaetae: (0) straight; (1) arc-like; (2) forked.
53. Body – placement of the longest macrochaetae: (0) VI abdomen; (1) V abdomen; (2) IV abdomen.
54. Body – cryptopygy: (0) absent; (1) present.
55. Body – bilobation of the last abdominal segment: (0) absent; (1) present.
56. Body – reticulations: (0) absent; (1) present.
57. Body – buttonhole structures: (0) absent; (1) present.
58. Body – tubercles between tergites of thorax and abdomen: (0) absent; (1) present.
59. Thorax – number of tubercles on the first tergite (excluding tubercles Dl): (0) tubercles absent; (1) 2 or 3 tubercles present; (2) 4 tubercles present.
60. Thorax – chaetae Di on the first tergite: (0) present; (1) absent.
61. Thorax – number of chaetae Di on the second tergite: (0) 4 or more chaetae; (1) 3 or less chaetae.
62. Thorax – number of tubercles on the second and third tergites (excluding tubercles L): (0) tubercles absent; (1) 4 tubercles present; (2) 6 tubercles present; (3) 8 tubercles present.
63. Thorax – tubercles De on the second and third tergites: (0) absent; (1) present and

not divided; (2) present and divided.

64. Thorax – tubercles Dl on the second and third tergites: (0) absent; (1) present and not divided; (2) present and divided.
65. Thorax – number of chaetae L on the second and third tergites: (0) 2 chaetae; (1) 3 chaetae; (2) more than 3 chaetae.
66. Thorax – position of sensillum p3 on the second and third tergites: (0) not moved forward and in row p; (1) moved forward and in row m.
67. Abdomen – supplementary s-chaetae on tergites I-III: (0) absent; (1) present.
68. Abdomen – number of tubercles on segments I–II (excluding tubercles L): (0) tubercles absent; (1) 4 tubercles present; (2) 6 tubercles present; (3) 8 tubercles present.
69. Abdomen – number of tubercles on tergite III (excluding tubercles L): (0) tubercles absent; (1) 4 tubercles present; (2) 5 tubercles present; (3) 6 tubercles present; (4) 8 tubercles present.
70. Abdomen – tubercles Di on tergites I–III: (0) absent; (1) present.
71. Abdomen – tubercles De on tergites I–III: (0) absent; (1) present.
72. Abdomen – tubercles Dl and L on tergites I–III: (0) absent; (1) present and separated; (2) present and fused.
73. Abdomen – fusion of abdominal tergites IV–VI: (0) absent; (1) present.
74. Abdomen – number of tubercles on tergite IV (excluding tubercles L): (0) tubercles absent; (1) 2 tubercles present; (2) 3 tubercles present; (3) 4 tubercles present; (4) 5 tubercles present; (5) 6 tubercles present.
75. Abdomen – tubercles Di on tergite IV: (0) absent; (1) present and separated; (2) present and fused along midline.
76. Abdomen – tubercles De on tergite IV: (0) absent; (1) present and separated; (2) present and fused with tubercles Dl; (3) present and fused with tubercles Dl and Di.
77. Abdomen – tubercles Di on tergite V: (0) absent; (1) present and separated; (2) present and fused along midline.
78. Abdomen – tubercles Di and De on tergite V: (0) absent; (1) present and separated; (2) present and fused.
79. Abdomen – length of segment V: (0) shorter than segment IV; (1) longer than segment IV.
80. Abdomen – number of tubercles on tergite VI: (0) tubercles absent; (1) 1 tubercle present; (2) 2 tubercles present.
81. Abdomen – unpaired chaeta or spine on tergite VI: (0) present; (1) absent.
82. Abdomen – spines on tergite VI: (0) present; (1) absent.
83. Abdomen – number of chaetae td on the ventral tube (on half of the TV): (0) 2 chaetae; (1) 3 chaetae; (2) 4 or more chaetae.
84. Abdomen – number of chaetae tp on the ventral tube (on half of the TV): (0) 2 or more chaetae; (1) 1 chaeta.
85. Abdomen – tenaculum: (0) present; (1) absent.
86. Abdomen – furca: (0) well developed with mucro; (1) reduced to two small rolls bearing chaetae; (2) reduced to unpaired roll bearing chaetae or without.
87. Abdomen – male ventral organ: (0) absent; (1) present.
88. Abdomen – number of chaetae Ve on half of sternite II: (0) 7 or less chaetae; (1) 8 or more chaetae.

89.	Abdomen – chaeta Ve1 on sternite II: (0) present; (1) absent.
90.	Abdomen – number of chaetae L on segment I: (0) 3 or less chaetae; (1) 4 or more chaetae.
91.	Abdomen – number of chaetae L on segment II: (0) 3 or less chaetae; (1) 4 or more chaetae.
92.	Abdomen – number of chaetae L on segment III: (0) 4 or less chaetae; (1) 5 or more chaetae.
93.	Abdomen – tubercle L on sternite IV: (0) absent; (1) present.
94.	Abdomen – tubercle L on sternite V: (0) absent; (1) present.
95.	Legs – tooth on claw: (0) present; (1) absent.
96.	Legs – number of chaetae on subcoxae2 of the second and third pair of legs: (0) 2 chaetae present; (1) 3 chaetae present.
97.	Legs – M chaeta on tibiotarsus: (0) present; (1) absent.
98.	Legs – number of chaetae on trochanters: (0) 6 or more chaetae; (1) 5 chaetae.
99.	Legs – elongated and clavate chaetae B4 and B5 on tibiotarsus: (0) present; (1) absent.
100.	Head – apical organ on hyaline plain of labium*: (0) present; (1) absent.
