# Supplementary material for: Is the Current Systematic Subdivision of the Subfamily Neanurinae (Collembola, Neanuridae) Still Valid? Testing the Monophyly and Phylogenetic Relationships of Currently Established Tribes of the Neanurinae
Source: Insects. 2024 Sep 5;15(9):672. doi: 10.3390/insects15090672 (PMC11432141; doi:10.3390/insects15090672)
Supplement: Supplementary file 1 [file insects-15-00672-s001.zip › Table S2. Matrix.pdf]

Table S2. Morphological data matrix used in the analyses.

Taxon	Characters
	1 1 1 1 1 1 1 1 1 1 2 2 2 2 2 2 2 2 2 2 3 3 3 3 3 3 3 3 3 3 4 4 4 4 4 4 4 4 4 4 5 5 5 5 5 5
	0 1 2 3 4 5 6 7 8 9 0 1 2 3 4 5 6 7 8 9 0 1 2 3 4 5 6 7 8 9 0 1 2 3 4 5 6 7 8 9 0 1 2 3 4 5 6 7 8 9 0 1 2 3 4 5
Friesea mirabilis	0 0 0 0 0 0 0 0 0 0 0 0 0 0 2 0 0 1 0 0 0 0 0 1 0 1 1 0 0 0 0 0 0 0 1 0 0 0 0 0 0 0 0 0 0 0 2 0 0 0 0 1 0 0 0 0 0
Pseudachorutes palmiensis	0 0 0 0 0 0 0 0 0 0 0 0 0 0 2 0 0 1 0 0 0 0 0 1 0 1 0 0 0 0 0 0 0 0 0 1 0 0 0 0 0 0 1 0 0 0 2 0 1 0 0 1 0 0 0 0 0
Morulodes serratus	1 0 2 1 2 0 1 1 1 1 1 2 1 1 1 1 3 0 0 0 1 1 1 0 1 1 1 1 1 0 0 0 0 1 0 0 1 0 0 0 1 0 0 1 2 3 0 1 0 0 1 0 1 2 1 1
Paranura sexpunctata	2 0 0 0 0 0 0 0 0 0 0 0 0 1 0 0 1 0 0 0 1 1 1 0 2 1 1 1 1 0 0 0 0 1 1 1 1 0 0 1 2 1 2 1 2 3 1 1 1 1 1 0 1 0 0 0
Nahuanura ce	3 0 0 0 0 0 1 2 2 2 0 0 0 2 0 0 4 1 1 0 1 1 1 0 2 1 1 1 1 1 0 0 0 1 1 0 1 0 0 0 2 2 1 1 2 ? ? 1 1 1 1 2 0 0 0 0
Oregonanura cascadensis	2 0 1 2 2 0 1 2 2 1 1 1 0 1 0 0 1 0 0 0 1 1 1 0 2 1 1 1 1 0 0 0 0 1 1 1 1 0 0 0 1 2 0 1 2 3 0 1 1 1 1 0 0 0 0 0
Sensillanura austriaca	3 1 2 1 2 0 1 1 1 1 1 1 0 2 0 0 1 0 0 0 1 1 1 0 2 1 1 1 1 0 1 0 0 1 1 1 1 0 0 1 1 1 0 1 2 3 0 1 1 1 1 2 0 1 0 1
Americanura mexicana	3 1 1 2 2 0 1 1 1 1 1 1 1 2 2 1 1 1 0 0 1 1 1 0 2 1 1 1 1 0 1 0 0 1 1 1 1 0 0 1 2 1 2 1 2 3 0 1 1 1 1 2 0 0 0 1
Palmanura mirabilis	3 0 1 2 2 0 0 0 1 0 1 1 1 2 2 1 0 1 1 0 1 1 3 1 0 1 1 1 1 0 1 0 0 1 1 1 1 0 0 1 2 1 2 1 2 3 0 1 1 1 1 2 1 2 0 1
Tabasconura tapijulapana	3 0 1 2 2 0 1 1 1 1 1 1 1 0 2 1 0 1 0 0 1 1 3 1 0 1 1 1 1 1 1 0 0 1 1 1 1 0 0 1 1 1 2 1 2 3 0 1 1 1 1 2 1 0 0 1
Honduranura centraliamericana	3 0 2 1 2 0 1 2 1 1 1 2 1 1 2 1 0 0 1 0 1 1 1 0 1 1 1 1 1 0 1 0 0 1 1 1 1 0 0 0 2 1 0 1 2 3 0 1 1 1 1 1 0 0 0 1
Coecoloba plumleyi	3 1 1 2 2 0 0 0 0 0 0 0 0 1 2 0 1 0 1 0 1 1 1 0 2 1 1 1 1 0 0 0 1 1 0 0 1 0 0 0 1 0 2 1 2 3 1 1 0 0 0 2 0 0 0 1
Hemilobella rounsevelli	2 0 2 1 2 1 0 1 1 1 1 1 1 1 0 0 2 0 0 0 1 1 1 0 1 1 1 1 1 0 0 0 0 1 1 1 1 0 0 0 2 2 2 1 2 3 1 1 1 1 1 2 0 1 0 1
Sulobella yoshii	2 0 0 0 0 0 0 0 1 0 0 0 0 1 0 0 1 0 0 0 1 1 0 0 2 1 1 1 1 0 0 0 ? 1 1 1 1 0 0 0 1 1 2 1 2 3 1 1 1 1 1 1 0 0 0 1
Telobella kemiri	2 0 0 0 0 0 0 0 1 0 1 1 1 1 2 0 1 0 0 1 1 1 1 0 2 1 1 1 1 0 0 0 ? 1 0 1 1 0 0 0 1 0 2 1 2 3 1 1 1 0 1 2 0 1 0 1
Yuukianura judithae	3 1 1 0 2 1 0 1 1 0 1 1 0 1 0 0 3 0 0 1 1 1 1 0 2 1 1 1 1 0 0 0 1 1 0 0 1 0 0 0 1 0 2 1 2 3 1 1 0 0 1 2 0 0 0 1
Paralobella breviseta	2 0 3 1 1 1 0 1 1 1 1 1 1 1 0 0 2 0 0 0 1 1 1 0 1 1 1 1 1 0 0 0 1 1 1 0 1 0 0 0 1 1 2 1 2 3 1 1 0 0 1 1 0 1 0 1
Lobellina weinerae	2 0 3 1 1 1 0 1 1 0 1 1 1 1 0 0 1 0 1 0 1 1 1 0 2 1 1 1 1 0 0 0 0 1 0 1 1 0 0 0 1 0 2 1 2 3 1 1 1 0 1 1 0 0 0 1
Neanura muscorum	2 0 2 2 1 0 1 1 1 1 1 1 1 2 2 0 1 0 0 0 1 1 1 0 2 1 1 1 1 0 0 0 0 1 1 1 1 0 0 0 1 1 0 1 2 3 0 1 1 1 1 0 0 0 0 1
Deutonura phlegrea	3 0 2 1 2 0 1 1 1 1 1 2 1 1 2 0 2 0 0 0 1 1 1 0 1 1 1 1 1 0 0 0 0 1 1 1 1 0 0 0 1 1 0 1 2 3 0 1 1 1 1 0 1 1 1 1
Thaumanura carolii	2 0 2 1 2 0 1 2 1 1 1 1 0 1 0 0 4 0 0 0 1 1 1 0 2 1 1 1 1 0 0 0 0 1 1 1 1 0 0 0 1 1 2 1 2 1 1 1 1 1 1 0 0 0 0 1
Monobella grassei grassei	3 0 2 1 2 0 1 2 2 2 1 2 2 1 2 0 4 0 0 0 1 1 1 0 1 1 1 1 1 0 0 0 0 1 1 1 1 0 0 0 1 1 0 1 2 3 0 1 1 1 1 1 1 1 0 1
Vietnura caerulea	3 0 1 2 2 0 1 1 2 1 1 2 1 0 2 1 0 1 1 1 1 1 3 1 0 1 1 1 1 0 0 0 0 1 1 1 1 0 0 0 1 1 0 1 2 3 0 1 1 1 1 0 1 1 1 1
Edoughnura rara	3 0 2 1 2 0 1 2 1 1 1 1 2 1 0 0 4 1 0 0 1 1 1 0 1 1 1 1 1 0 0 0 0 1 0 1 1 0 0 0 2 0 1 1 2 3 0 1 0 0 1 0 0 1 0 1
Xylanura oregonensis	2 0 2 1 2 0 2 0 1 1 1 1 0 1 0 0 1 0 0 0 1 1 1 0 2 1 1 1 1 0 0 0 0 1 1 1 1 0 0 0 1 1 0 1 2 3 0 1 1 1 1 0 0 0 0 1
Ghirkanura chernovae	3 0 2 1 2 0 1 2 1 1 1 1 2 1 2 1 0 1 1 0 1 1 2 0 0 1 1 1 1 0 0 0 0 1 1 1 1 0 0 1 2 1 ? ? ? ? ? 1 1 1 1 2 2 1 0 0
Intricatonura fjellbergi	2 0 1 2 2 0 1 1 1 1 1 2 1 1 0 1 1 1 1 1 1 1 3 1 0 1 1 1 1 0 0 0 0 1 1 1 1 0 1 1 2 1 1 1 2 3 1 1 1 1 1 0 0 1 1 1
Paravietnura notabilis	3 0 1 2 2 0 1 1 2 1 1 2 1 0 2 1 0 1 0 0 1 1 3 1 0 1 1 1 1 0 0 0 0 1 1 1 1 0 0 1 2 2 1 1 2 3 0 1 1 1 1 0 1 2 1 1
Australonura grossi	3 1 2 1 2 0 1 1 1 1 1 1 2 1 2 0 3 1 0 0 1 1 1 0 2 1 1 1 1 0 0 0 0 1 1 1 1 0 0 0 2 2 2 1 2 3 0 1 1 1 1 1 0 0 0 1
Himalmeria gurung	3 1 1 2 2 0 1 2 2 2 1 1 1 0 2 0 4 1 0 0 1 1 1 0 2 1 1 1 1 0 0 0 0 1 1 1 1 0 0 0 1 2 2 1 2 1 0 1 1 1 1 1 0 0 0 0
Zelandanura bituberculata	3 1 1 2 2 0 1 1 2 1 1 1 2 1 2 1 1 0 0 0 1 1 1 0 2 1 1 1 1 0 0 0 0 1 1 ? 1 0 0 ? ? 1 2 1 2 3 0 1 1 1 1 2 0 1 0 0
Caledonura tillierae	3 1 1 2 2 0 1 2 2 2 1 2 1 0 2 1 0 1 1 0 1 1 3 1 0 1 1 1 1 0 0 0 0 1 1 ? 1 0 0 ? ? 2 2 1 2 3 0 1 1 1 1 2 0 1 0 1
Cameronura delamarei	3 1 1 2 2 0 1 2 1 1 1 2 1 1 2 1 1 0 0 1 1 1 1 0 2 1 1 1 1 0 0 0 0 1 0 1 1 0 0 0 2 0 2 1 2 3 1 1 1 1 1 1 0 1 1 1
Ectonura lata	3 1 2 1 2 0 2 1 2 1 1 1 0 0 2 1 1 0 0 0 1 1 1 0 2 1 1 1 1 0 0 0 0 1 1 0 1 0 0 1 1 1 1 1 2 3 1 1 1 1 1 2 1 0 0 1
Galanura agnieskae	3 1 0 0 0 0 2 1 1 0 0 0 0 1 0 0 1 0 1 0 1 1 1 0 2 1 1 1 1 0 1 1 0 1 1 1 1 0 0 0 1 1 0 0 1 0 0 1 1 1 1 2 0 0 0 0
Paleonura epiphytica	3 1 0 0 0 1 0 1 1 1 0 0 0 1 0 0 1 0 0 0 1 1 1 0 2 1 1 1 1 0 0 0 0 1 1 0 1 0 0 0 1 1 2 1 2 3 0 1 1 1 1 2 0 1 0 1
Pronura pomorskii	3 1 1 2 2 0 1 2 2 2 1 2 1 2 2 0 4 0 0 0 1 1 1 0 2 1 1 1 1 0 0 0 0 1 1 1 1 1 1 1 2 2 2 1 2 3 1 1 1 1 1 2 0 1 1 0
Vitronura mascula	3 1 1 2 2 1 0 1 1 0 1 1 1 2 2 1 0 1 1 1 1 1 3 1 0 1 1 1 1 0 0 0 0 1 1 1 1 0 0 1 2 2 2 1 2 3 0 1 1 1 1 2 0 2 0 1
Bilobella carpatica	3 1 1 2 2 0 1 1 1 1 1 2 1 0 0 0 0 0 0 1 1 1 1 0 2 1 1 1 1 0 1 1 0 1 1 0 1 0 0 0 1 1 0 1 2 3 0 1 1 1 1 2 0 1 1 1

Itanura brasiliensis	3 1 2 1 2 0 1 1 1 1 1 1 0 1 2 1 1 1 0 0 1 1 1 0 2 1 1 1 1 0 0 0 0 1 1 0 1 0 0 0 2 1 2 1 2 3 0 1 1 1 1 2 0 1 1 1

Table S2. Continued

**Taxon**	**Characters**
	1<br>5 5 5 5 6 6 6 6 6 6 6 6 6 6 7 7 7 7 7 7 7 7 7 7 8 8 8 8 8 8 8 8 8 8 9 9 9 9 9 9 9 9 9 9 0<br>6 7 8 9 0 1 2 3 4 5 6 7 8 9 0 1 2 3 4 5 6 7 8 9 0 1 2 3 4 5 6 7 8 9 0 1 2 3 4 5 6 7 8 9 0
Friesea mirabilis	0 0 0 0 0 0 0 0 0 0 0 0 0 0 0 0 0 0 0 0 0 0 0 0 0 0 0 0 0 0 0 0 0 0 0 0 0 0 0 1 0 1 1 0 1
Pseudachorutes palmiensis	0 0 0 0 0 0 0 0 0 0 0 0 0 0 0 0 0 0 0 0 0 0 0 0 0 0 1 0 0 0 0 1 0 1 0 0 0 0 0 0 1 0 0 1 1
Morulodes serratus	1 0 0 2 0 1 2 1 1 1 1 0 1 1 1 1 1 0 2 2 2 1 2 0 2 1 1 1 1 1 1 0 0 1 0 1 0 1 1 1 1 0 0 0 0
Paranura sexpunctata	0 0 0 0 0 0 1 0 0 0 1 1 0 0 0 0 0 0 0 0 0 0 0 0 0 1 1 1 1 1 2 0 0 1 0 0 0 0 0 1 0 1 1 1 1
Nahuanura ce	1 0 0 0 0 1 0 0 0 1 1 0 0 0 0 0 0 0 0 0 0 2 2 0 1 1 1 1 1 1 2 0 0 1 1 1 0 0 0 1 0 1 0 1 1
Oregonanura cascadensis	1 0 0 1 0 1 2 1 1 1 1 0 1 1 1 1 2 0 1 1 3 2 1 1 1 1 1 1 1 1 2 0 0 1 1 1 1 0 0 1 0 0 0 1 1
Sensillanura austriaca	1 0 0 1 0 1 2 1 1 1 1 0 1 1 1 1 1 0 5 1 1 2 1 0 2 1 1 1 1 1 2 0 0 0 0 0 0 0 0 1 0 0 1 1 1
Americanura mexicana	1 0 0 1 1 1 2 1 1 1 1 0 1 1 1 1 1 0 5 1 1 1 1 0 2 1 1 1 1 1 2 0 0 0 0 0 0 0 0 1 0 1 1 1 1
Palmanura mirabilis	1 0 0 1 1 1 2 1 1 1 1 0 1 1 1 1 1 0 3 1 2 1 2 0 2 1 1 1 1 1 2 0 0 1 0 0 0 0 0 1 ? 1 ? 1 1
Tabasconura tapijulapana	1 0 0 1 1 1 2 1 1 1 1 0 1 1 1 1 1 0 3 1 2 1 1 0 2 1 1 1 1 1 2 0 0 1 0 0 0 0 1 1 ? 1 ? 1 1
Honduranura centraliamericana	1 0 0 1 1 1 2 1 1 1 1 0 1 1 1 1 1 0 5 1 1 1 2 0 1 1 1 1 1 1 2 0 0 1 1 1 0 1 1 1 0 1 0 1 1
Coecoloba plumleyi	0 1 0 0 0 1 1 1 1 1 0 1 1 1 0 1 1 0 1 0 1 1 1 0 2 1 1 1 1 1 2 0 0 ? 0 0 0 0 0 0 ? 0 ? 1 1
Hemilobella rounsevelli	0 0 0 2 0 1 2 1 1 1 1 1 1 1 1 1 1 0 5 1 1 1 1 0 2 1 1 1 1 1 2 0 0 0 1 1 1 0 1 0 0 0 0 1 1
Sulobella yoshii	0 0 0 0 0 0 1 1 0 2 0 1 1 1 1 1 1 0 5 0 1 0 1 0 2 1 1 1 1 1 2 0 0 1 1 1 1 1 1 0 ? 0 0 1 1
Telobella kemiri	0 0 0 0 0 1 2 1 1 1 0 1 1 1 1 1 1 0 5 1 1 1 1 0 2 1 1 1 1 1 2 0 0 1 0 0 0 1 1 0 ? 0 0 1 1
Yuukianura judithae	0 1 0 0 0 1 1 1 1 1 0 1 1 1 0 1 1 0 1 0 2 1 2 0 2 1 1 1 1 1 2 0 0 1 1 1 1 1 1 1 0 1 0 1 1
Paralobella breviseta	1 0 0 2 0 1 2 1 1 1 1 1 1 1 1 1 1 0 5 1 1 1 1 0 2 1 1 1 1 1 2 0 0 1 1 1 1 0 1 0 0 0 0 0 1
Lobellina weinerae	0 0 0 2 0 1 2 1 1 1 0 0 1 1 1 1 1 0 5 1 1 1 1 0 2 1 1 1 1 1 2 0 0 1 1 1 0 1 1 0 0 0 0 1 1
Neanura muscorum	1 0 0 2 0 1 2 1 1 1 1 0 1 1 1 1 1 0 5 1 1 1 1 0 2 1 1 1 1 1 2 0 0 0 0 0 0 0 0 1 0 0 0 1 1
Deutonura phlegrea	1 0 0 2 0 1 2 1 1 1 1 0 1 1 1 1 1 0 5 1 1 2 1 0 2 1 1 1 1 1 2 0 0 0 0 0 0 0 0 1 0 0 0 1 1
Thaumanura carolii	1 0 0 1 0 1 3 2 1 1 1 0 1 1 1 1 1 0 5 1 1 1 1 1 1 1 1 1 1 1 2 0 1 0 1 1 1 0 0 1 1 0 0 0 1
Monobella grassei grassei	1 0 0 2 0 1 1 1 1 1 1 0 1 1 1 1 1 0 2 2 1 2 2 0 2 1 1 1 1 1 2 0 0 0 0 0 0 0 0 1 0 0 0 1 1
Vietnura caerulea	1 0 0 2 0 1 2 1 1 1 1 0 1 1 1 1 1 0 5 1 1 2 1 0 2 1 1 1 1 1 2 0 0 0 0 0 0 0 0 1 0 0 0 1 1
Edoughnura rara	1 0 0 1 0 1 2 1 1 1 1 0 1 1 1 1 1 0 4 2 1 2 1 0 2 1 1 1 1 1 2 0 0 0 0 0 0 0 0 1 0 1 0 1 1
Xylanura oregonensis	1 0 0 0 0 1 2 1 1 1 1 0 1 1 0 1 1 0 3 1 1 2 1 0 2 1 1 1 1 1 2 0 0 0 0 0 0 0 0 1 0 0 0 1 1
Ghirkanura chernovae	1 0 0 1 0 1 2 1 1 1 1 0 1 1 1 1 1 0 4 2 1 2 1 0 2 1 1 1 1 1 2 0 0 0 0 0 0 0 0 1 0 0 0 1 1
Intricatonura fjellbergi	1 0 0 2 0 1 3 1 2 1 1 0 1 1 1 0 1 0 3 1 0 1 2 0 2 1 1 1 1 1 2 0 0 0 0 0 0 0 0 1 0 1 0 1 1
Paravietnura notabilis	1 0 0 2 0 1 2 1 1 1 1 0 1 1 1 1 1 0 5 1 1 1 2 0 2 1 1 1 1 1 2 0 0 1 0 0 0 0 0 1 0 0 0 1 1
Australonura grossi	1 0 0 2 0 1 2 1 1 1 1 0 1 1 1 1 1 0 5 1 1 2 1 0 1 1 1 1 1 1 2 0 0 0 0 0 0 0 0 1 0 1 0 1 1
Himalmeria gurung	0 0 0 2 0 1 2 1 1 1 1 0 1 1 1 1 2 1 5 1 1 2 2 0 1 1 1 1 1 1 2 0 0 1 1 1 1 0 0 1 ? 1 ? 1 1
Zelandanura bituberculata	1 0 0 2 0 1 2 1 1 1 1 0 1 1 1 1 1 0 4 2 1 1 2 0 1 1 1 1 1 1 2 0 0 0 0 0 0 1 0 1 ? 1 ? 1 1
Caledonura tillierae	1 0 0 1 1 1 2 1 1 1 1 0 1 1 1 1 1 0 5 1 1 2 1 0 2 1 1 1 1 1 2 0 0 1 1 0 1 0 1 1 ? 1 0 1 1

Cameronura delamarei	0 0 0 1 0 1 2 1 1 1 1 0 1 1 1 1 2 1 3 1 2 1 1 0 2 1 1 1 1 1 2 0 0 0 0 0 0 0 0 1 ? 1 ? 1 1
Ectonura lata	0 0 0 0 0 1 1 1 1 1 1 0 1 1 0 1 1 0 3 0 1 0 1 0 2 1 1 1 1 1 2 1 0 0 0 0 0 0 0 1 ? 1 ? 1 1
Galanura agnieskae	1 0 0 0 0 1 2 1 1 1 1 0 1 1 1 0 1 0 5 1 1 1 1 0 1 1 1 1 1 1 2 0 0 0 0 0 0 0 0 1 0 1 1 1 1
Paleonura epiphytica	0 0 0 1 0 1 1 1 1 1 1 0 1 1 0 1 1 0 5 1 1 1 1 0 2 1 1 1 1 1 2 0 0 1 0 0 0 0 0 1 0 0 0 0 1
Pronura pomorskii	1 0 1 2 0 1 2 1 1 1 1 0 1 1 1 1 1 0 4 2 1 1 2 0 1 0 1 1 1 1 2 0 0 1 0 0 0 0 0 1 0 1 1 1 1
Vitronura mascula	1 0 0 2 0 1 2 1 1 1 1 0 1 1 1 1 1 0 5 1 1 1 1 0 2 1 1 1 1 1 2 1 0 1 0 0 0 0 1 1 0 1 0 1 1
Bilobella carpatica	1 0 0 1 1 1 2 1 1 1 1 0 1 1 1 1 1 0 3 1 2 2 2 0 1 1 1 1 1 1 2 0 0 1 0 0 0 1 0 1 0 0 0 1 1
Itanura brasiliensis	0 0 0 0 0 1 1 1 1 1 1 0 1 1 0 1 1 0 3 0 1 1 1 0 2 1 1 1 1 1 2 0 0 0 0 0 0 0 0 1 ? 1 ? 1 1
