# Supplementary material for: Is the Current Systematic Subdivision of the Subfamily Neanurinae (Collembola, Neanuridae) Still Valid? Testing the Monophyly and Phylogenetic Relationships of Currently Established Tribes of the Neanurinae
Source: Insects. 2024 Sep 5;15(9):672. doi: 10.3390/insects15090672 (PMC11432141; doi:10.3390/insects15090672)
Supplement: Supplementary file 1 [file insects-15-00672-s001.zip › Table S3. List of morphological apomorphies.pdf]

Table S3. Morphological apomorphies. Character number from „Character list" followed by state in parentheses; non-homoplasious changes indicated in bold.

Node	Characters
42	**74(2)**, 75(2)
43	11(2), 24(1), 52(1), 53(1)
44	1(0), 51(0)
45	**12(1)**, 14(2)
46	70(1)
47	35(1), 77(2)
48	2(2), 3(1), 4(2), 10(1), 11(1)
49	**9(1)**, 55(1), **71(1)**, 80(2)
50	1(1), **40(2)**, **63(1)**, **64(1)**, **68(1)**, **69(1)**, **72(1)**, **74(5)**, **75(1)**, **76(1)**
51	51(2), **61(1)**, **65(1)**, 80(1)
52	**20(1), 21(1)**, **24(2)**, **26(1)**, **27(1)**, **28(1)**, **36(1)**, 40(2), **43(1)**, **44(2)**, **45(3)**, **48(1)**, 49(1), **66(1)**, **81(1)**, **83(1)**, **84(1)**, **85(1)**, **86(2)**
53	7(2), 12(0), 59(1), 79(1), 80(1)
54	14(0), 90(1), 91(1), 92(1)
55	0(2), 77(1)
56	17(1), 39(1), 80(2)
57	30(1), 59(1), **60(1)**
58	2(1), 3(2), 77(1)
59	40(2), 42(2), 80(1), 97(1)
60	17(1), 40(2)
61	**22(3)**, **23(1)**, 24(0)
62	11(2), 42(0), 93(1)
63	0(3), 1(1), 2(1), 4(2), 35(0), 48(0), **57(1)**, 70(0), 74(1)
64	12(0), 62(1), 75(0)
65	2(0), 3(0), 4(0), 5(0), 7(0), 59(0)
66	9(0), 34(0), 41(0), 66(0), 93(1)
67	**2(3)**, 4(1), 49(0), 89(1)
68	5(1), 6(0), 67(1), 94(1), 95(0)
69	7(2), 12(2)
70	8(2), 52(1), 97(0)
71	1(0), 42(1), 51(0), 54(1)
72	19(1), 59(2), 60(0)
73	30(0), 53(1)
74	11(1), 17(1), 40(2), 59(1), 74(4)
75	51(1), 56(0), 72(2), **73(1)**
76	No synapomorphies
77	74(4), 75(2)
78	14(2), 15(1), 75(0)

Taxon	Characters
Americanura mexicana	98(1)
Australonura grossi	12(2), 16(3), 17(1), 51(1)
Bilobella carpatica	14(0), 19(1), 31(1), 35(0), 40(1), 53(1), 54(1), 74(3), 76(2), 77(2), 97(0)
Caledonura tillierae	7(2), 8(2), 9(2), 77(2), 90(1), 92(1)
Cameronura delamarei	11(2), 19(1), 34(0), 46(1), 54(1), 59(1), 74(3), 76(2), 80(2)
Coecoloba plumleyi	3(2), 8(0), 14(2), 18(1), **50(0)**, 90(0), 91(0), 93(0), 94(0)
Deutonura phlegrea	No autapomorphies
Ectonura lata	8(2), 13(0), 39(1), 46(1), 52(1), 77(0), 87(1)
Edoughnura rara	14(0), 34(0), 41(0), 48(0), 49(0), 97(1)
Galanura agnieskae	30(1), 31(1), 35(1), 43(0), 44(1), 45(0), 70(1)
Ghirkanura chernovae	15(1), 16(0), 18(1), **22(2)**, 24(0), 39(1), 51(2), 55(0)
Hemilobella rounsevelli	40(2), 41(2)
Himalmeria gurung	9(2), 13(0), 16(4), 17(1), 40(1), 45(1), 77(2), 90(1), 91(1), 92(1)
Honduranura centraliamericana	1(0), 2(2), 3(1), 7(2), 18(1), 24(1), 51(1), 90(1), 91(1), 94(1)
Intricatonura fjellbergi	0(2), 14(0), 16(1), 38(1), 46(1), 62(3), **64(2)**, 71(0), 74(3), 76(0)
Itanura brasiliensis	17(1), 40(2), 53(1), 54(1)
Lobellina weinerae	18(1), 67(0)
Monobella grassei grassei	8(2), 9(2), 51(1), 62(1)
Morulodes serratus	**0(1)**, **14(1)**, 15(1), 34(0), 35(0), 41(0), 48(0), 49(0), 53(2), 76(2), 77(1), **86(1)**, 89(1), 91(1), 93(1), 94(1), 96(1), 99(0), **100(0)**
Nahuanura ce	9(2), 16(4), 17(1), 29(1), 41(2), 42(1), 90(1), 91(1)
Neanura muscorum	3(2), 4(1), 13(2)
Oregonanura cascadensis	2(1), 3(2), 8(2), 41(2), 55(0), 72(2), 74(1), **76(3)**, 77(2), 89(1)
Paleonura epiphytica	5(1), 53(1), 59(1), 99(0)
Palmanura mirabilis	6(0), 7(0), 9(0), 53(2), 78(2)
Paralobella breviseta	35(0), 48(0), 99(0)
Paranura sexpunctata	35(1), 39(1), 46(1), 52(1), 67(1)
Paravietnura notabilis	18(0), 19(0), 53(2)
Pronura pomorskii	9(2), 11(2), 13(2), 16(4), **37(1)**, 38(1), 46(1), 54(1), **58(1)**, 81(0)
Sensillanura austriaca	13(2), 30(1), 39(1), 53(1), 98(1)
Sulobella yoshii	**22(0)**, 34(1), 41(1), 49(1), 61(0), 64(0), **65(2)**, 77(0)
Tabasconura tapijulapana	29(1), 40(1)
Telobella kemiri	14(2), 19(1), 90(0), 91(0)
Thaumanura carolii	16(4), 45(1), 62(3), **63(2)**, **88(1)**, 96(1), 99(0)
Vietnura caerulea	39(0), 40(1), 42(0), 77(2)
Vitronura mascula	5(1), 6(0), 9(0), 53(2), 87(1)
Xylanura oregonensis	0(2), 1(0), 7(0), 51(0)
Yuukianura judithae	5(1), 7(1), 16(3), 19(1), 76(2), 78(2), 95(1), 97(1)
Zelandanura bituberculata	12(2), 93(1)
